# Correlates of local safety-related concerns in a Swedish Community: a cross-sectional study

**DOI:** 10.1186/1471-2458-9-221

**Published:** 2009-07-08

**Authors:** Agneta Kullberg, Nadine Karlsson, Toomas Timpka, Kent Lindqvist

**Affiliations:** 1 Linköping University, Department of Medical and Health Sciences, SE-581 83 Linköping, Sweden

## Abstract

**Background:**

Crime in a neighbourhood has been recognized as a key stressor in the residential environment. Fear of crime is related to risk assessment, which depends on the concentration of objective risk in time and space, and on the presence of subjective perceived early signs of imminent hazard. The aim of the study was to examine environmental, socio-demographic, and personal correlates of safety-related concerns at the local level in urban communities. The specific aim was to investigate such correlates in contiguous neighbourhoods in a Swedish urban municipality.

**Methods:**

A cross-sectional study design was used to investigate three neighbourhood settings with two pair-wise conterminous but socially contrasting areas within each setting. Crime data were retrieved from police records. Study data were collected through a postal questionnaire distributed to adult residents (*n *= 2476) (response rate 56%). Composite dimensions of perceived residential safety were derived through a factor analysis. Logistic regression analysis was used to examine associations between high-level scores of the three safety-related dimensions and area-level crime rate, being a victim of crime, area reputation, gender, age, education, country of birth, household civil status and type of housing.

**Results:**

Three composite dimensions of perceived residential safety were identified: (I) structural indicators of social disorder; (II) contact with disorderly behavior; and (III) existential insecurity. We found that area-level crime rates and individual-level variables were associated with the dimensions structural indicators of social disorder and existential insecurity, but only individual-level variables were associated with the dimension contact with disorderly behavior. Self-assessed less favorable area reputation was found to be strongly associated with all three factors. Being female accorded existential insecurity more than being a victim of crime.

**Conclusion:**

We have identified environmental, socio-demographic, and personal correlates of safety-related concerns in contiguous neighbourhoods in a Swedish community. The results of this study suggest that residents' self-assessed area reputation is an important underlying mechanism of perceived safety. We also found a difference in crime rates and safety-related concerns between areas with blocks of flats compared with small-scale areas although the neighbourhoods were close geographically.

## Background

Since the mid-19th century, public health practitioners have understood that people's residential environment and housing conditions influence health outcomes [1,2]. Crime is among those factors suggested to be most strongly related to health in the housing environment, both by direct exposure and indirectly by residents' perception of feeling unsafe [3,4]. Previous research suggests that several factors influence residents' perceived safety in a neighbourhood. These factors can be divided into three main themes: crime experiences and fear of crime, demographic characteristics, and the design and quality of the residential environment [5-9].

### Crime experiences and fear of crime

Crime in a neighbourhood has been recognized as a key stressor in the residential environment, when measured in absolute numbers of reported crimes in the neighbourhood and as residents' personal experience of being a crime victim. Fear of crime has also been found to have a significant negative influence on residential quality, and may have a negative impact on residents' mental health comparable to crime itself [10,11].

Fear of crime is related to risk assessment, which depends on the concentration of objective risk in time and space, and on the presence of subjective perceived early signs of imminent hazard [12]. Prolonged safety-related worry and fear of crime is suggested to yield behaviour modification as it potentially decreases physical activity and limits the residents' personal freedom. A consequence of safety-related worry may therefore be 'time-space inequalities' with implications for physical and mental health [13,14].

However, crime is an elusive factor with many facets. Violent crime in the immediate environment does not seem to have a great deal in common with every day offences, such as car theft or damage, in relation to perceived safety. Another aspect is the way the local newspaper communicates crime in specific neighbourhoods. Newspaper headlines about local crime may result in attitudes that divide neighbourhoods into desirable and undesirable areas.

Stories retold by neighbours who have been victims of crime themselves may result in negative attitudes, and increase the perception of isolation among residents [15,16]. There is thus a community-based contagious process affecting residents' perceived safety in specific areas through the influence of reports in the media and/or others around them.

### Demographic characteristics

Earlier studies have reported that individual variables such as gender, age, social and economic resources influence the potential risk of becoming a crime victim and fear of crime. Even though women are subject to lower crime victim rates than men, they are found to fear crime more [17,18]. Similarly, the elderly fear crime to a higher degree than young adults, even though they have the lowest risk of becoming a victim themselves [19]. Further, Halpern [20] found that to be unmarried or socially disconnected from conventional society in the area where you live, the more likely you are to become a victim of crime.

At neighbourhood level, the proportion of residential heterogeneity, level of education, mobility rate and density of the neighbourhood were found to be associated with perception of safety of the people living there [21,22].

### Design and quality of the residential environment

Newman [23] presented the model of 'defensible space' in planning and argued for a correlation between design and function of the built environment and crime rates. He observed that in small-scale neighbourhoods in which the residents have good surveillance and control of shared outdoor space, the possibility of achieving lower factual crime rates and better maintenance is higher than in neighbourhoods without the possibility of this informal control. Newman also observed that residents who had to share a common entrance and staircase to reach their dwelling reported more perceived insecurity and a lower sense of belonging to the neighbourhood.

Land-use mix is another aspect that has been suggested to influence crime rates. For instance, non-residential property, such as shops, car-parking and public spaces, has been found to increase the number of crimes [24]. There is also evidence that visible signs such as litter and graffiti in an area communicate anti-social behaviour and how seriously the risk of becoming a crime victim has to be taken. The perception that one's neighbourhood is unsafe is likely a constant psychological irritant [25,26]. Several studies have suggested that perceptions of safety and fear of crime play an important role in explaining area differences in health through emotional and behavioural responses on neighbourhood quality [27-29].

Factors such as the appearance of the neighbourhood and the reputation of the area have thus been found to impact on residential well-being [30,31] but few studies have focused on how these characteristics influence safety-related concerns. Due to the fact that neighbourhoods are embedded in a community context in which reputations are developed, the rumours that evolve may affect residents' sense of belonging in different neighbourhoods in different ways. If a neighbourhood is held to have a specific reputation, this will influence the way its residents are perceived by others. Depending on the character of the reputation, this influence could have either a positive or negative effect on an individual's self-esteem [32,33].

### Study aims

The aim of the study was to examine environmental, socio-demographic, and personal correlates of safety-related concerns at the local level in urban communities. The specific aim was to investigate such correlates in contiguous neighbourhoods in a Swedish urban municipality.

## Methods

### Study setting

The study was undertaken at the local level in a Swedish urban municipality (population 42,000 in 2005). Six housing areas with a total of 6300 residents (aged 20–79 years) were investigated. The selection of the housing areas was made after consultation with municipality officials in order to choose areas geographically recognized by local people. As a basis, the municipal administrative office provided a map showing the areas geographically defined for statistical purposes [34].

The six housing areas selected were geographically located two-by-two, i.e. two areas were spatially contiguous to each other in three different neighbourhood settings. The areas were given fictitious names indicating the type of property. Setting Alpha incorporates area *Alpha-house *(detached houses) and *Alpha-flat *(blocks of flats); setting Beta incorporates area *Beta-mix *(mixed tenure and types of houses) and *Beta-flat *(blocks of flats); and setting Gamma incorporates area *Gamma-house *(detached houses) and *Gamma-flat *(blocks of flats).

The proportion of non-residential land-use was higher in the three areas with blocks of flats. Within each of the three settings there was a small local community centre with both public and private services such as a grocery store, day-care centre, bus transport and a common public school, all with good access irrespective of the living area within the setting. Within each setting, the residents also shared a fair-sized green space for recreation.

The characteristics of the areas based on recorded data are presented in Table 1. The population in each of the two areas within the three settings contrasted with the other demographically and socio-economically. The residents in the small-scale areas were more socially advantaged and more residentially stable than the residents in areas with blocks of flats. Motor-vehicle density was higher in the small-scale areas (Table 1).

**Table 1:** Structural features of the housing areas studied^a^

	Setting Alpha		Setting Beta		Setting Gamma	
	
Variable	Alpha-house	Alpha-flat	Beta-mix	Beta-flat	Gamma-house	Gamma-flat
Total population^b^	1423	1918	1857	1680	794	1364
Residents by type of property^a^						
Blocks of flats (%)	10 (0.7)	1918 (100)	761 (41)	1675 (99.7)	0	1262 (93)
Detached houses (%)	1413 (99.3)	0	1096 (59)	5 (0.3)	794 (100)	97 (7)
Period of construction^a^	1966–1975	1966–1970	1951–1960	1966–1970	1971–75	1961–1965
Resident turnover (%)^c^	107 (7.5)	363 (18.1)	272 (14.4)	289 (17.4)	45 (5.8)	262 (18.4)
Motor-vehicle density (no./1000 inhabitants)^d^	430	270	380	303	464	291
Mean for whole municipality (index 100):						
Gainfully employed 20–64 years^e^	114	81	102	80	104	85
Disposable income >20 years^e^	131	77	95	66	122	75
Housing allowance^f^	97	113	79	89	93	100
Social allowance^g^	48	109	84	124	27	131
>12 years in school (%)^b^	264 (26.1)	126 (9.5)	281 (21.5)	142 (12)	98 (16.9)	98 (9.7)
High-income residents (%)^h^	222 (22)	44 (3.3)	202 (15.4)	35 (3)	81 (14)	39 (4)

^a^Data source: Statistics Sweden.

^b^Date 30 September 2005.

^c^Residents' turnover 1 January 2005 to 31 December 2005.

^d^Date 31 December 2004.

^e^INKOPAK, 2004.

^f^Housing allowance for families with children (bostadsbidrag) as well as for pensioners (bostadstillägg).

^g^The social allowance should give a reasonable standard of living.

^h^High income defined as ≥ 300,000 Swedish crowns (SEK)/year in 2004. US$1 = SEK6.6; EUR1 = SEK9.0; date 30 December 2004.

Registered data on crime rates for each housing area were collected from the local police office (Table 2). The data on reported crime were grouped into five categories according to the Swedish penal code: (a) crime against life and health; (b) crime against freedom and serenity; (c) theft and robbery; (d) crime of damage; (e) other crimes. Information for 3 years (2003–2005) was included to increase area stability for reported crime. In settings Alpha and Gamma, the mean total crime rates in the areas with blocks of flats were five to six times those in the small-scale areas. In setting Beta only a comparatively small difference in crime rates was observed between Beta-flat and the mixed tenure area, Beta-mix. Theft and robbery were the most frequently reported crimes in all areas. The rates of reported violence and other crimes against personal safety were ten (setting Alpha) to four (setting Beta) times as high in the areas with blocks of flats than in the small-scale or mixed areas within those two settings. The residents in the small-scale areas and in the mixed area, Alpha-house, Beta-mix and Gamma-house, had organized Neighbourhood Watch programmes against crime and signposts in those areas were visible.

**Table 2:** Police reported crime in the housing areas studied^1^

	Setting Alpha		Setting Beta		Setting Gamma	
	
	Alpha-house	Alpha-flat	Beta-mix	Beta-flat	Gamma-house	Gamma-flat
Mean number of police reported crime/1000 inhabitants per year (%)						
(a) Crime against life and health (homicide, manslaughter, maltreatment)	0.7 (2.4)	10.6 (5.8)	2.3 (3.1)	10 (9.8)	0.9 (2.5)	7.8 (5.2)
(b) Crime against freedom and serenity (unlawful threat, molest)	2.6 (8.9)	23.5 (12.9)	3.3 (6.2)	12.4 (12.2)	4.2 (12.7)	21.1 (12.7)
(c) Theft and robbery	16.7 (58.5)	88.3 (48.5)	36.1 (48.7)	49.3 (48.2)	20.4 (60.8)	81.2 (48.7)
(d) Crime of damage	5.1 (17.9)	30.8 (16.9)	13.7 (18.5)	10.8 (10.6)	3.4 (10.1)	18.6 (11.2)
(e) Other crimes	3.5 (12.2)	28.8 (15.9)	17.5 (23.5)	19.6 (19.2)	4.7 (13.9)	36.9 (22.1)

Total	28.6 (100)	182 (100)	72.9 (100)	102.1 (100)	33.6 (100)	165.6 (100)

^1^Data source: Local police office. Based on the years 2003–2005.

### Study population

A cross-sectional study was conducted among the residents living in the six neighbourhoods. A sample was randomly selected from the census records by Statistics Sweden and residents aged 20–79 years were invited to participate in a survey. The questionnaire was sent by post in October 2005. The questionnaire was followed up by a reminder 4 weeks later and a further questionnaire was posted to non-respondents. A total of 1390 residents participated, accounting for 56% of the 2476 individuals who received the questionnaire.

### Measurements

As there is no consensus on the term 'safety-related concerns' [8,35], alternative concepts describing the term are derived from a set of relevant variables.

#### Safety-related concerns

The items of perceived *neighbourhood disorder *were adapted from the Swedish Living Conditions survey [36] (items a-f) or original for this study (g), but developed by the WHO [37]. The participants were asked to assess each question using a five-point Likert scale: to what extent they perceived (a) damage and/or graffiti, (b) littering, (c) car theft, (d) disturbance from neighbours. Participants were asked to respond using a measurement scale from 'Yes, very annoying' to 'None'. The respondents were also asked to what extent they perceived (e) tobacco smoking and (f) consumption of alcohol or use of other drugs to be an annoyance in their housing area. The respondents were also asked to estimate (g) how often their sleep was disturbed because of street noise or noisy neighbours.

Questions about *general safety and fear of crime *were asked to measure perceived safety based on previously used questions (with minor modifications). The item, 'Do you feel safe or insecure in your housing area in the daytime?' and the item 'Do you feel safe or insecure in your housing area during the evening and at night?' was intended to capture time and space aspects of perceived safety in the residential environment. To measure *fear of crime *the question, 'Do you refrain from going outdoors because of fear of crime?' was used as in previous research [11,37-39].

#### Environmental and personal correlates

The respondents were categorized according to *geographic housing area *and neighbourhood setting. A question was asked on whether the residents had personal *experience as a victim of crime *during the last 12 months within the housing area. The reply option was 'yes' or 'no' and the item was original but modified from Statistics Sweden [36]. The participants were also asked to rate their perceived *area reputation *using a Likert scale: from 'very good' to 'very bad'. This question is original, but developed from studies in the west of Scotland [40].

#### Area-level crime

The area-level crime rates were represented as the mean number of police-reported crimes per 1000 inhabitants per year (year 2003–2005) according to the penal codes presented in Table 2.

#### Demographic characteristics

*Age *(in 2005) was grouped in three categories: 'young' as 20–34 years, 'middle-age' as 35–64 years and 'elderly' as 65–79 years of age. *Education *was grouped by length of education. The participants were classified into three groups: 'low educated' as 1–9 years in school, 'middle educated' as 10–12 years in school and 'high educated' as more than 12 years in school. *Country of birth *was categorized as 'born in Sweden' or 'elsewhere'. *Household civil status *was categorized as 'family household' when living with a spouse or significant other and/or children or 'single household' when living alone. *Type of housing *was categorized as 'small house' or 'flat'.

### Statistical method

A factor analysis was performed to derive simplified dimensions of safety-related concerns from the set of variables measuring several aspects of perceived safety. The primary variables included in the analyses were the extent of graffiti, car theft, litter, disturbance by neighbours, disrupted sleep, disturbance by tobacco smoking and alcohol consumption, sense of safety during the day, evening and night, and fear of crime. The extraction method was principal component analysis and an orthogonal rotation was performed with varimax and Kaiser normalization using the option of replacement of missing values with the mean in SPSS [41].

For each dimension of perceived safety identified, the factor scores were computed for each respondent. Due to the skewness of the distribution of the factor scores (*p *< 0.001) [42], these were dichotomised by the upper quartile, for use as input variables in subsequent statistical analyses.

A total of 1097 persons with no missing values on risk item and condition variables were included in the subsequent analyses. Logistic regressions with robust estimates of standard errors were performed to examine associations between derived concepts of safety-related concerns and area- and individual-level variables. Clustering effects within each neighbourhood were taken into account by calculating robust estimates of standard errors in Stata. The multivariate adjusted odds ratios with 95% confidence intervals were estimated for total area crime rate (*area-level characteristic*), having been a victim of crime, area reputation, gender, age, education, country of birth, household civil status and type of housing (small house vs flat) (*individual characteristics*). A level of 5% was considered to be statistically significant.

#### Ethical aspects

The study was approved by the Regional Committee for Research Ethics at Linköping University.

## Results

### Non-responder characteristics

The response rate varied between areas and was the highest in area Gamma-house (detached houses), 158/231 (68%), and lowest in area Gamma-flat (blocks of flats), 175/391 (45%). External dropout analysis showed no statistically significant differences in gender among the responders compared to the study population in five of the six areas, but in area Alpha-house more females and fewer males responded than expected. The responders were older than the study population in three areas (Alpha-house, Beta-mix, Beta-flat). The group of 'middle educated' (10–12 years) responders was underrepresented in three areas (Alpha-house, Alpha-flat, Beta-mix) but there was no statistically significant difference in the distribution of country of birth between the responders and the study population (Table 3).

**Table 3:** Demographic characteristics of the survey respondents and study population in the housing areas studied

	Setting Alpha		Setting Beta		Setting Gamma	
	
	Alpha-house n (%)	Alpha-flat n (%)	Beta-mix n (%)	Beta-flat n (%)	Gamma-house n (%)	Gamma-flat n (%)
**Gender distribution**						
Respondents						
Male	108 (40.1)	101 (42.1)	139 (44.4)	108 (46)	68 (43.0)	77 (44.0)
Female	141 (52.4)	125 (52.1)	163 (52.1)	111 (47.2)	85 (53.8)	82 (46.9)
Partial drop-outs: non- respondent gender	20 (7.4)	14 (5.8)	11 (3.5)	16 (6.8)	5 (3.2)	16 (9.1)
Total	269	240	313	235	158	175
Study population						
Male	514 (50.8)	646 (48.9)	632 (48.3)	569 (48.2)	280 (48.2)	507 (51.6)
Female	497 (49.2)	676 (51.1)	677 (51.7)	611 (51.8)	301 (51.8)	476 (48.4)
Total	1011	1322	1309	1180	581	983
*p*-value	0.035*	0.246	0.480	0.766	0.409	0.461
**Age distribution**						
Respondents						
20–34 years	21 (7.8)	56 (23.3)	51 (16.3)	59 (25.1)	25 (15.8)	35 (20)
35–64 years	170 (63.2)	132 (55)	173 (55.3)	98 (41.7)	87 (55.1)	95 (54.3)
65–79 years	74 (27.5)	50 (20.8)	84 (26.8)	74 (31.5)	45 (28.5)	40 (22.9)
Partial drop-outs: non-respondent age	4 (1.5)	2 (0.8)	5 (1.6)	4 (1.7)	1 (0.6)	5 (2.9)
Total	269	240	313	235	158	175
Study population						
20–34 years	131 (13)	406 (30.7)	321 (24.5)	362 (30.7)	97 (16.7)	280 (28.5)
35–64 years	654 (64.7)	695 (52.6)	712 (54.4)	529 (44.8)	328 (56.5)	515 (52.4)
65–79 years	226 (22.4)	221 (16.7)	276 (21.1)	289 (24.5)	156 (26.9)	188 (19.1)
Total	1011	1322	1309	1180	581	983
*p*-value	0.027*	0.051	0.004**	0.044*	0.897	0.08
**Country of birth**						
Respondents						
Born in Sweden	253 (94.1)	165 (68.8)	283 (90.4)	186 (79.1)	136 (86.1)	115 (65.7)
Born elsewhere	15 (5.6)	75 (31.3)	30 (9.6)	48 (20.4)	20 (12.7)	56 (32)
Partial drop-outs: non-respondent country of birth	1 (0.4)	0	0	1 (0.4)	2 (1.3)	4 (2.3)
Total	269	240	313	235	158	175
Study population						
Born in Sweden	939 (92.9)	870 (65.8)	1188 (90.8)	891 (75.5)	503 (86.6)	612 (62.3)
Born elsewhere	72 (7.1)	452 (34.2)	121 (9.2)	289 (24.5)	78 (13.4)	371 (37.7)
Total	1011	1322	1309	1180	581	983
*p*-value	0.378	0.375	0.852	0.192	0.843	0.212
**Years in school**						
Respondents						
1–9 years	82 (30.5)	98 (40.8)	88 (28.1)	94 (40)	55 (34.89)	71 (40.6)
10–12 years	78 (29)	74 (30.8)	106 (33.9)	76 (32.3)	62 (39.2)	63 (36)
≥ 12 years	71 (26.4)	26 (10.8)	82 (26.2)	31 (13.2)	26 (16.5)	19 (10.9)
Partial drop-outs: non-respondent years in school	38 (14.1)	42 (17.5)	37 (11.8)	34 (14.5)	15 (9.5)	22 (12.6)
Total	269	240	313	235	158	175
Study population						
1–9 years	266 (26.3)	564 (42.7)	365 (27.9)	505 (42.8)	171 (29.4)	438 (44.6)
10–12 years	481 (47.6)	632 (47.8)	663 (50.6)	533 (45.2)	312 (53.7)	447 (45.5)
≥ 12 years	264 (26.1)	126 (9.5)	281 (21.5)	142 (12)	98 (16.9)	98 (10)
Total	1011	1322	1309	1180	581	983
*p*-value	0.001**	0.017*	0.001**	0.116	0.064	0.493
No. of questionnaires delivered (*N *= 2510)	413	518	514	443	231	391
Drop-out on account of deceased or moved (*N *= 34)						
Respondent rate (*N *= 1390, 56.1%)	269 (65.1)	240 (46.3)	313 (60.9)	235 (53)	158 (68.4)	175 (44.8)

*χ^2 ^test significant at *p *< 0.05. **χ^2 ^test significant at *p *< 0.01.

### Composite dimensions of deficient residential safety

Three composite dimensions of residential safety-related concerns were identified in the factor analysis: 'structural indicators of social disorder', 'contact with disorderly behaviour' and 'existential insecurity' (Table 4). Each composite dimension was associated with different sets of modifying items and conditions.

**Table 4:** Factor loadings for three dimensions of perceived safety (*n *= 1390)

	Derived factor^a^		
	
	I. Structural indicators of social disorder	II. Contact with disorderly behaviour	III. Existential insecurity
Percentage of variation explained	40.2	12.1	10.3
Cronbach alpha	0.78	0.78	0.65
Factor loadings			
Graffiti	0.83		
Car theft	0.74		
Litter	0.73		
Disturbing neighbours		0.83	
Disturbed night rest		0.82	
Tobacco smoking		0.58	
Alcohol consumption		0.54	
Sense of safety in evening and night			0.74
Fear of crime			0.73
Sense of safety in daytime			0.73

^a^Factor loadings < 0.5 are not represented.

### Structural indicators of social disorder

Area-level crime rates were associated with reporting by the residents of structural indicators of social disorder (OR 1.010, CI 1.007–1.013) (Table 5). Residents who estimated their area reputation as less good were more than twice as likely to report concerns about structural indicators of social disorder (OR 2.86, CI 2.13–3.84). For females, the odds ratio of reporting such concerns was lower (OR 0.78, CI 0.65–0.94). Residents born abroad reported concerns about structural indicators of social disorder to a lower extent (OR 0.68, CI 0.55–0.84) than residents born in Sweden. Living in a flat was associated with concerns in this dimension (OR 1.37, CI 1.01–1.84), but having been a victim of crime, age, and household civil status were not associated (Table 5).

**Table 5:** Logistic regression analysis with robust estimates of standard errors for three dimensions of perceived safety

Variables		Structural indicators of social disorder			Contact with disorderly behaviour			Existential insecurity		
	N (%)	Exposed cases, *n* (%)	Adjusted OR^a^	95% CI	Exposed cases, *n* (%)	Adjusted OR^a^	95% CI	Exposed cases, *n* (%)	Adjusted OR^a^	95% CI

**Area-level characteristics**										
Total area crime^b^	-	-	1.01***	1.01–1.01	-	1.00	0.99–1.00	-	1.00**	1.00–1.01
**Individual characteristics**										
Experience of crime										
No (0)	982 (89.5)	225 (22.9)	1		222 (22.6)	1		218 (22.2)	1	
Yes (1)	115 (10.5)	51 (44.3)	1.61	0.82–3.18	49 (42.6)	1.61*	1.03–2.51	35 (30.4)	1.42	0.85–2.37
Area reputation										
Good (0)	416 (37.9)	41 (9.9)	1		39 (9.4)	1		53 (12.7)	1	
Not very good (1)	681 (62.1)	235 (34.5)	2.86***	2.13–3.84	232 (34.1)	2.91***	2.12–4.00	200 (29.4)	2.67***	1.64–4.35
Gender										
Male	507 (46.2)	139 (27.4)	1		131 (25.8)	1		54 (10.7)	1	
Female	590 (53.8)	137 (23.2)	0.78**	0.65–0.94	140 (23.7)	0.93	0.79–1.10	199 (33.7)	4.54***	3.21–6.43
Age (years)										
20–34	214 (19.5)	64 (29.9)	1		76 (35.5)	1		55 (25.7)	1	
35–64	619 (56.4)	166 (26.8)	1.36	0.96–1.92	153 (24.7)	1.00	0.77–1.32	117 (18.9)	0.80	0.58–1.09
65–79	264 (24.1)	46 (17.4)	0.87	0.51–1.47	42 (15.9)	0.51***	0.38–0.70	81 (30.7)	1.72**	1.20–2.46
Education										
1–9 years	427 (38.9)	97 (22.7)	1		94 (22.0)	1		115 (26.9)	1	
10–12 years	433 (39.5)	131 (30.3)	1.40*	1.01–1.94	129 (29.8)	1.27	0.79–2.05	95 (21.9)	0.95	0.68–1.31
≥ 12 years	237 (21.6)	48 (20.3)	1.20	0.83–1.74	48 (20.3)	1.27	0.69–2.34	43 (18.1)	0.82	0.53–1.27
Country of birth										
Born in Sweden	909 (82.9)	218 (24.0)	1		209 (23.0)	1		204 (22.4)	1	
Born elsewhere	188 (17.1)	58 (30.9)	0.68***	0.55–0.84	62 (33.0)	1.04	0.74–1.47	49 (26.1)	1.02	0.69–1.51
Household civil status										
Family household	831 (75.8)	197 (23.7)	1		168 (20.2)	1		184 (22.1)	1	
Single household	266 (24.2)	79 (29.7)	0.85	0.61–1.20	103 (38.7)	1.57***	1.28–1.94	69 (25.9)	0.94	0.61–1.47
Type of housing										
Small house	453 (41.3)	52 (11.5)	1		35 (7.7)	1		79 (17.4)	1	
Flat	644 (58.7)	224 (34.8)	1.37*	1.01–1.84	236 (36.6)	5.58***	3.06–10.17	174 (27.0)	0.85	0.59–1.23

OR, odds ratio; CI, confidence interval. *p < 0.05;**p < 0.01;***p < 0.001.

^a^ORs are adjusted for all other variables in the table.

^b^Total mean number of police-reported crimes/1000 inhabitants per year.

### Contact with disorderly behaviour

The odds ratio for reporting contact with disorderly behaviour was more than five times higher for residents living in flats than for those living in detached houses (OR 5.58, CI 3.06–10.17) (Table 5). Personal experience of crime during the last 12 months increased the likelihood of being concerned about disorderly behaviour in the residential area to 1.61 (CI 1.03–2.51). Such concerns were also associated with living in a neighbourhood with estimated less favourable reputation (OR 2.91, CI, 2.12–4.00). The odds ratio for reporting contact with disorderly behaviour was significantly lower for the elderly (65–79 years) (OR 0.51, CI 0.38–0.70). There were no associations between area-level crime, gender, education, country of birth and contact with disorderly behaviour, but for single households the odds ratio was 1.57 times higher (CI 1.28–1.94) than for family households.

### Existential insecurity

The odds ratio of reported existential insecurity (feeling insecure in the residential area during the day or in the evening or at night, and fear of crime) in the neighbourhood was associated with area-level crime (OR 1.003, CI 1.001–1.006) but not significantly associated with being a crime victim during the last 12 months (OR 1.42, CI 0.85–2.37). Residents who thought their area reputation was less good had more than twice the odds ratio (OR 2.67, CI 1.64–4.35) for experiencing existential insecurity in the neighbourhood. Existential insecurity in the neighbourhood was reported four times more often by female residents (OR 4.54, CI 3.21–6.43) compared with males, and being elderly (65–79 years) increased the odds ratio for perceived existential insecurity to 1.72 (CI 1.20–2.46) times that of younger residents. Education, country of birth, household civil status, and type of housing were not associated with existential insecurity in the neighbourhood (Table 5).

## Discussion

In our study design, an attempt was made to cover several complementary dimensions of perceived safety in the residential environment. The three derived dimensions captured almost two-thirds of the total item variance. As could be expected, the dimension 'structural indicators of social disorder' was associated with the area-level of crime and to living in a flat. Previous research [24] suggested that a land-use structure with large non-residential space, such as in the present areas with blocks of flats, increases crime rates. A corresponding association between factual crime rates and perceived structural indicators of social disorder could be observed in the areas with detached houses and little non-residential space, where the residents assessed the occurrence of threatening physical signs as less serious; the crime rates were also relatively low. It is possible that signs of disorder in the residential environment give rise to safety-related concerns among residents, especially in combination with local reports of violence, assaults or rape communicated through neighbours or the media. In areas where reports of crime and signs of disorder co-exist, a process of stigmatization could develop giving an area the reputation of being a dangerous place [43,44]. In the classic theory of 'broken windows', Wilson and Kelling [45] suggest that signs of neighbourhood disorder are perceived as a warning sign of low reciprocity. More recent research has indicated that physical signs of social disorder indicate neighbourhood deterioration, trigger general insecurity among residents and stimulate outward migration [46-49].

We observed a similar pattern of clear differentiation between residents in different types of housing for the dimension 'contact with disorderly behaviour'. Residents living in flats reported more severe problems with disturbance from neighbours, alcohol consumption, and smoking, and their sleep was disturbed more frequently because of noise in the residential environment. Contact with this sort of disorderly behaviour could be interpreted as a lack of the normal inhibitors of incivilities and crime, which produces insecurity. If such a pattern lasts for a prolonged in time, residents may suffer from the consequences of safety-related concerns in the living environment as a source of accumulated stress with negative impact on well-being and indirectly on health [50]. The two composite dimension; 'structural indicators of social disorder' and 'contact with disorderly behaviour' both reflect the construct *collective efficacy *introduced by Sampson [51], and defined as the linkage of social control and cohesion that reproduces the norms for behaviour in neighbourhood environments. Several studies demonstrate that where the rules of behaviour are unclear, residents will experience mistrust and safety-related concerns. Poor social and economic resources and residential instability in a neighbourhood have been found to be strongly associated with violence mediated by low collective efficacy [52-54]. This pattern could be applicable to the present study; in the areas with comparatively high violence-related crime rates (blocks of flats), the levels of education and income were lower, and the density of the residents and the mobility rate were higher than for areas with low violence rates (small-scale housing areas). The occurrence of violence in the immediate environment may generate a situation where it is harder for residents to build trustworthy bonds with their neighbours [54]. If social disturbances are reported and communicated regularly from particular areas in the local media, this negative information will accumulate over time and have a negative impact on the image of a specific area and partly mould its identity among its residents.

In accordance with previous studies, we found that female gender was strongly associated with the composite dimension 'existential insecurity' (to feel unsafe during the day or in the evening or at night and fear of crime). It is possible that this dimension reflects an individual vulnerability, in contrast to the other concepts, which is derived from determinants in physical space and the built environment to a higher extent [55]. It is also known that persons who are the object of violence or threats are often closely related to the criminal [18]. Arguments have been made on the need for local safety promotion programs to take different perceptions of males and females into account [35]. To spend time outdoors in the evening and at night has been found to be correlated with an increased risk of being robbed or mugged [56]. Theft and robbery were the most prevalent types of crime in all areas in our study. Thus, area-level crime was associated with the existential insecurity dimension of safety-related concerns, but having been a victim of crime and type of housing were not. These findings could be interpreted to mean that existential insecurity is a more complex issue than area of residence, type of housing and victimization. On the other hand, in this context it is also interesting to observe that country of birth, level of education and living alone were not important for perceived 'existential insecurity'.

We found that area-level crime rate and individual-level variables were associated with the composite dimensions 'structural indicators of social disorder' and 'existential insecurity', but only individual-level variables were associated with the dimension 'contact with disorderly behaviour'.

We investigated whether there was an association between the residents' self-estimated area reputation and safety-related concerns. Our data show that all three derived safety-related dimensions of safety-related concerns ('structural indicators of social order', 'contact with disorderly behaviour' and 'existential insecurity ') were strongly influenced by whether the residents perceived themselves to live in a residential environment with a less favourable reputation. This study indicates that self-assessed area reputation is an invisible factor that strongly influences the sense of insecurity among residents. Why area reputation is a mechanism that has strong consequences for perceived safety among residents is not investigated here but it is possible that reputation is related to social dimensions in the residents' immediate environment. In this regard, clues can be found in studies suggesting that the occurrence of crime and incivilities is associated with the development of perceptions that one's neighbourhood is a 'bad place' [57,58]. Another plausible clue is that if the estimated reputation is an essential part of the lives of residents, mental images of the area could either strengthen the attachment to the neighbourhood if you live in a 'good place' or weaken the attachment if you live in a 'bad place'. The mental images the residents have of their neighbourhood are likely to have serious consequences on the community spirit of their neighbourhood, leading to either a positive or negative situation in which residents increase or reduce their willingness to come together. A less favourable reputation develops a process of stigmatization as a 'bad area', leading to an ongoing migration process in which residents who are able, move away from the area. Consequently the people with lesser ability and resources remain. In contrast, the reputation of a desirable 'good area' is attractive and implies a safe and secure place in which to live.

Living alone was found to be positively associated with 'contact with disorderly behaviour' when compared with residents living in families. This may be in line with earlier studies that suggested that living alone could lead to social isolation and thereby a more vulnerable situation [20], which likely also generates anxiety.

The three small-scale areas in this study had implemented Neighbourhood Watch programs. The lowest crime rates were recorded from these areas and the residents in detached houses reported the highest perceived safety. The rationale behind Neighbourhood Watch programs is to contribute to lower crime rates and to a perception of safety in a neighbourhood through increased reciprocity, e.g. looking after strangers and property and improved informal social control. However, a pre-existing high social capital in the neighbourhood seems to be a prerequisite for implementation of a successful Neighbourhood Watch program [18,20,59]. In Sweden, the majority of Neighbourhood Watch programs are implemented in wealthy small-scale areas [60]. Therefore, if Neighbourhood Watch programs are to become an effective method of building safer neighbourhoods, our results suggest that the design of these programs need to be adjusted to make them suitable for introduction in areas with blocks of flats.

### Study limitations

There are important limitations that have to be considered when trying to draw conclusions from this study. A methodological limitation is the cross-sectional design, which does not allow causal inferences. Given this study design, and the fact that the variables in the field of study are strongly interrelated, it is not possible to determine if a negative area reputation is a cause or a consequence of low perceived safety. Area reputation appears to be part of a complex pathway through which the effects of crime on perceived safety are mediated. However, our results are only indicative on this matter.

A number of variables that could contribute to perceived safety were not included in this study, e.g. health indicators, individual and contextual social capital, length of residence, and objective neighbourhood characteristics (concentrated affluence, unemployment rate, outdoor conditions such as lighting in public areas, etc.) [33,61,62].

In previous housing area studies based on postal questionnaires in Scotland [63] and Australia [64], the participation rates were around 50%, similar to the 56% achieved in this study. The pattern of non-response was slightly skewed in the present study according to gender, age and education for some areas. Some findings may therefore have been somewhat overestimated, due to the fact that the participants included a higher proportion of women (Alpha-house) and older persons (Alpha- house, Beta-mix, Beta-flat) compared with the total area population.

## Conclusion

We have identified environmental, socio-demographic, and personal correlates of safety-related concerns in contiguous neighbourhoods in a Swedish community. The results indicate that self-assessed area reputation was important for all dimensions of safety-related concerns among the residents. We also found a difference in crime rates and safety-related concerns between areas with blocks of flats compared with small-scale areas, although these neighbourhoods were close geographically with a shared local centre.

Our findings suggest that the implementation of modern Swedish housing policy targeting social integration has resulted in inequalities in safety-related concerns among residents. Future research on whether Neighbourhood Watch programs are an effective way to promote safer neighbourhoods is warranted, as well as the development of methods to implement these programs in areas with blocks of flats.

## Competing interests

The authors declare that they have no competing interests.

## Authors' contributions

AK, KL, TT planned the study. AK was the main author of this paper and was involved in all steps to create the manuscript. NK designed the statistical analysis. All authors read drafts of the manuscript, made comments and suggestions to improve the text before they agreed to submit it for publication.

## Pre-publication history

The pre-publication history for this paper can be accessed here:


